# The Inflammation–Thrombosis Genetic Axis in Abdominal Aortic Aneurysm: A Shared Roadmap to Precision Medicine

**DOI:** 10.1055/a-2936-0175

**Published:** 2026-09-03

**Authors:** Yanfei Yang, Yuhang Liu, Yuexin Chen

**Affiliations:** 1 Department of Vascular Surgery, Peking Union Medical College Hospital 34732 Chinese Academy of Medical Sciences and Peking Union Medical CollegeBeijingChina; 2 36674 School of Basic Medical Sciences, Dalian Medical UniversityDalianLiaoningChina

**Keywords:** abdominal aortic aneurysm, inflammation and thrombosis, genetic susceptibility, genome-wide association study, shared genetic signatures

## Abstract

Abdominal Aortic Aneurysm (AAA) is characterized by persistent inflammation, extracellular matrix loss, and intraluminal thrombus formation, yet the genetic links among these processes remain incompletely defined. Here, we examine evidence that genetic variation can influence inflammatory and thrombotic responses at the same time. Findings from genome-wide association studies are considered alongside transcriptomic, proteomic, single-cell, epigenetic, and experimental data. Particular attention is given to candidate genes and signaling networks relevant to AAA susceptibility, enlargement, and rupture. The available evidence supports a model in which inherited susceptibility alters the balance between vascular inflammation, coagulation, fibrinolysis, and wall repair. However, many reported loci still lack functional confirmation, and most genetic data come from populations of European ancestry. Defining the causal variants and the cells in which they act will be necessary before these findings can be used for individualized screening or treatment.

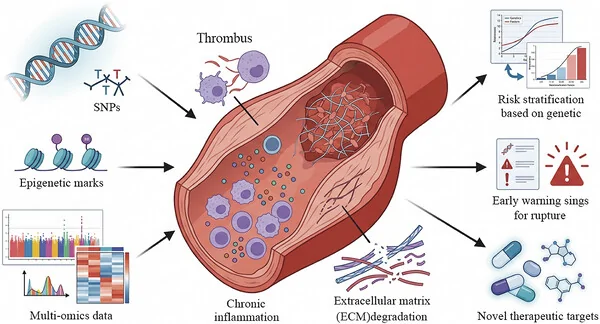

## Introduction

Abdominal Aortic Aneurysm (AAA) is a degenerative disorder of the abdominal aorta that predominantly affects older adults and can be fatal after rupture. Its pathology is not confined to a single arterial layer. Loss of medial smooth muscle cells, disorganization of elastin and collagen, and remodeling of the adventitia develop together as the aneurysm enlarges. 1 2 Chronic leukocyte infiltration and intraluminal thrombus (ILT) are also common, although their relative contributions vary among patients and across different regions of the same aneurysm. 3

Inflammatory cells are abundant in AAA tissue, but their effects depend on cellular state and location. Activated macrophages and lymphocytes release cytokines and proteases, including matrix metalloproteinases (MMPs), that favor vascular smooth muscle cell (VSMC) loss and extracellular matrix (ECM) breakdown. 4 MMP-2 and MMP-9 can cleave elastin and collagen and thereby weaken the load-bearing structure of the aortic wall. 5 Single-cell RNA sequencing (scRNA-seq) has added further resolution by identifying interferon-inducible monocytes/macrophages (IFNICs). In experimental AAA, this population is associated with cGAS-STING and JAK-STAT activation, type I interferon signaling, and pyroptosis. 6 Adaptive immunity is also involved: T cells, B cells, and tertiary lymphoid structures have been associated with aneurysm diameter and mural thrombus burden. 7 These observations indicate substantial immune heterogeneity rather than a uniform inflammatory response.

ILT formation occurs in a distinct hemodynamic and endothelial environment. Disturbed flow exposes subendothelial material, promotes platelet activation, and facilitates coagulation. 8 Signaling through glycoprotein VI (GPVI), the major platelet collagen receptor, may therefore affect more than clot formation; experimental evidence links GPVI-dependent platelet activity to inflammation and aortic wall remodeling. 8 The thrombus itself is biologically active and provides a site where leukocytes, platelets, proteases, and red blood cell products accumulate. Consequently, inflammation can favor thrombosis, whereas products released within the thrombus can further damage the underlying wall. 9

Genetic variation may influence several points along this inflammation–thrombosis interface and partly explain why AAA develops or progresses in some individuals but not in others. High-throughput genetic and molecular studies now make it possible to move beyond isolated candidate genes and ask whether particular variants act through specific vascular or immune-cell programs. 10 This review examines familial and population evidence, genome-wide association study (GWAS) and sequencing results, multiomics and single-cell studies, and functional experiments relevant to this question. We also consider what is currently required—and what remains uncertain—before polygenic risk scores (PRSs), pharmacogenomics, or epigenetic markers can inform AAA care.

### Familial Aggregation and Genetic Epidemiological Evidence

Extensive epidemiological studies have confirmed that AAA exhibits significant familial aggregation. A whole-aorta computed tomography (CT) screening study of relatives of AAA patients revealed a significantly higher prevalence than in age-matched general populations, providing direct imaging evidence. 11 A large-scale UK Biobank prospective cohort study using PRSs demonstrated that individuals with high genetic risk scores have a significantly increased risk of developing AAA. 12 Twin and family-based studies have estimated that genetic factors account for approximately 70–77% of AAA risk variance. 13 This substantial heritability provides a solid foundation for linkage analysis, candidate gene association studies, and GWAS.

### Major Technical Platforms and Strategies for Genetic Signature Identification

GWAS has become the predominant approach for identifying common genetic variants associated with AAA. A Million Veteran Program GWAS analyzed approximately 18 million DNA variants and identified 14 novel AAA risk loci, bringing the total known significant loci to 24. 14 UK Biobank GWAS identified novel loci near *LINC01021, ATOH8* , and *JAK2* , implicating inflammatory pathways. 15 A transnational meta-analysis uncovered 141 independent association signals, including 97 previously unreported loci. 16

Whole-genome sequencing has identified rare variants with high effect sizes, such as those involved in vitamin D signaling, cholesterol metabolism, and DNA repair. 17 Multiomics integrative analysis revealed significant genetic correlations between AAA and 21 cardiometabolic traits, with cholesterol metabolism and inflammation as the most prominent shared pathways. 18 Proteomic Mendelian randomization suggested that reduced levels of Kit ligand and Neogenin may be causal factors for AAA. 19 The scRNA-seq has delineated AAA molecular pathology at single-cell resolution, confirming the pivotal role of *SORT1* in VSMCs. 20 *SORT1* (sortilin) in VSMCs further regulates phenotype switching, the senescence-associated secretory phenotype, and cardiovascular calcification, thereby maintaining vascular homeostasis; its dysregulation contributes to VSMC dysfunction and atherosclerotic remodeling. 21 22 Methodologically, integrating scRNA-seq and emerging spatial transcriptomics with GWAS fine-mapping enables mapping of risk variants to specific cell types and microanatomical niches within the aneurysmal wall, providing a powerful framework to prioritize causal genes and interpret GWAS signals at single-cell and spatial resolution. 20 23

### Genes Associated with Innate Immunity and Inflammatory Signaling Pathways

**Fig. 1:**
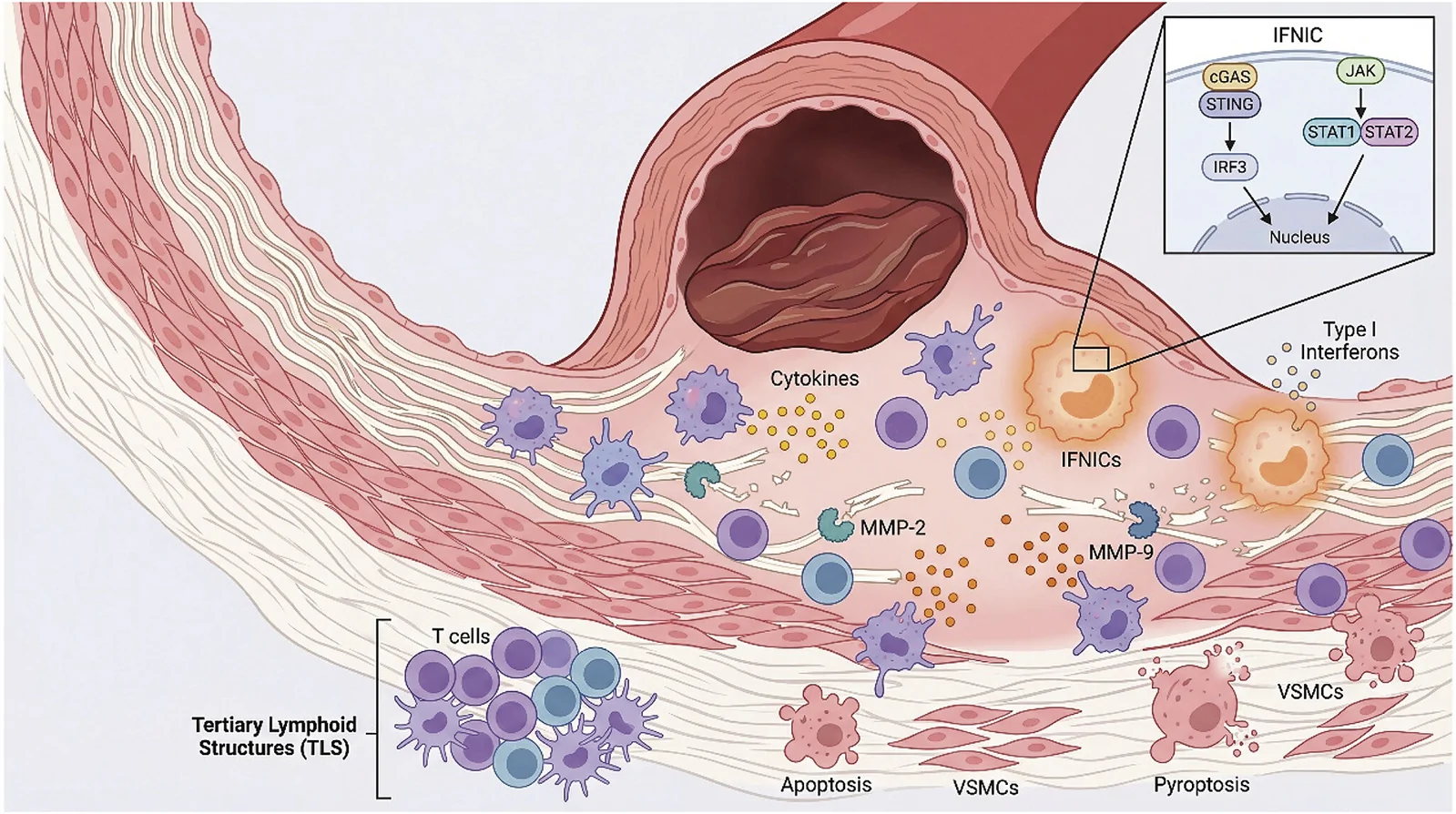
Proposed model of *SETDB2* -mediated epigenetic regulation in AAA. Interferon-β induces *SETDB2* expression via *JAK/* *STAT* signaling; *SETDB2* deposits *H3K9me3* on *TIMP* promoters, reducing TIMP expression and thereby enhancing MMP activity, ECM degradation, and AAA progression. (Source: Created in BioRender. Yang Y. (2026). https://app.biorender.com/illustrations/6a00a9126e7723ac703c5591?slideId=8c1bca9a-e593-49b8-b261-5ac2b802ec32 ). AAA, abdominal aortic aneurysm; ECM, extracellular matrix; IFNIC, interferon-inducible monocyte/macrophage; MMP, matrix metalloproteinase; VSMC, vascular smooth muscle cell.

Widespread dysregulation of inflammation-related gene expression is observed in AAA tissues. Layer-specific transcriptomics revealed 5,889 and 2,701 differentially expressed genes in the media and adventitia, respectively, with significant upregulation of immune-related gene sets. 24 In the angiotensin II-induced ApoE ^−/−^ ((apolipoprotein E knockout)) mouse AAA model, an *IFNIC* subpopulation driven by *cGAS* *-STING* and *JAK* *-STAT* pathways was identified. Myeloid cell-specific deletion of Sting1 or Ifnar1 significantly reduced AAA incidence and aortic diameter. 6 Notably, IFNIC-like interferon-driven macrophage subsets have also been detected in human AAA tissue by single-cell transcriptomics, supporting the translational relevance of this subpopulation beyond murine models. 20 The histone methyltransferase *SETDB2* (SET domain bifurcated histone lysine methyltransferase 2) is upregulated in monocytes/macrophages of human and murine AAA tissues via *JAK/* *STAT* signaling. 25 *SETDB2* catalyzes *H3K9me3* (trimethylation of histone H3 at lysine 9) at the *TIMP1–3* promoters, suppressing their transcription; tissue inhibitors of metalloproteinases ( *TIMPs* , including *TIMP1* ) are the endogenous inhibitors of MMPs that safeguard ECM integrity, so their epigenetic silencing removes a critical brake on ECM degradation and MMP activity. Macrophage-specific *SETDB2* deletion restored *TIMP* expression and protected mice from AAA formation 25 ( Fig. 1 ).

### Genes Associated with Extracellular Matrix Degradation and Proteases

Progressive ECM degradation is a hallmark of AAA. The exosome-related gene *FOSB* is upregulated in AAA and increases MMP-2/MMP-9 expression in VSMCs while suppressing contractile phenotype markers ( *α-* *SMA* (alpha-smooth muscle actin), *SM22α* (smooth muscle 22 alpha, also known as transgelin or TAGLN)). 26 *BAF60a* , an *SWI/* *SNF* (SWItch/Sucrose Non-Fermentable chromatin-remodelling complex) chromatin remodeling complex subunit, is upregulated in AAA lesions; VSMC-specific BAF60a deletion prevents AAA formation by attenuating inflammation and ECM degradation, partly through downregulating cathepsin S (CTSS). 27 Weighted gene coexpression network analysis (WGCNA) further identified upregulation of *Ctsa, Ctsc* , and *Ctsw* in AAA. 28 Functional evidence supports a role for the cathepsin family in AAA: Macrophage-derived *CTSS* promotes AAA progression, whereas cathepsin K ( *CTSK* ) gene disruption was reported not to affect murine aneurysm formation, indicating subtype-specific and context-dependent contributions that warrant further dissection. 27 29 Notably, *Ephx2* (encoding soluble epoxide hydrolase) is markedly elevated in the livers of AAA mice; hepatocyte-specific *Ephx2* deletion or pharmacological inhibition prevents AAA by reducing hepatic-derived inflammatory mediators such as complement C3 and serum amyloid A, 30 revealing a liver–aorta crosstalk.

### Genes Associated with the Coagulation and Anticoagulation Systems

Polymorphisms in coagulation factor genes ( *FII* , *FV* , *FVIII* , *FIX* ), anticoagulant proteins (protein C, protein S, antithrombin), and fibrinogen have been investigated for their influence on ILT susceptibility. 31 The factor V Leiden and prothrombin G20210A mutations have yielded inconsistent findings in AAA, highlighting the importance of genetic background in the coagulation pathway. 32 Thrombin, the central coagulation enzyme, converts fibrinogen to fibrin and amplifies coagulation but also exerts anticoagulant activity: Under physiological conditions, thrombin binds thrombomodulin on endothelial cells and activates protein C, generating activated protein C that inactivates FVa and FVIIIa and exerts cytoprotective effects 33 34 ; this dual pro-/anticoagulant role may become imbalanced in the AAA inflammatory microenvironment. In addition, the tissue factor (TF) pathway is physiologically restrained by tissue factor pathway inhibitor (TFPI), which limits the TF–FVIIa complex and FXa activity, and reduced TFPI activity may favor thrombosis in inflammatory vascular disease. 35 ILT from AAA patients is enriched in platelet-associated transcripts, suggesting close interplay between platelet activation and coagulation. 36 Notably, the ABO blood group locus has been reproducibly associated with AAA in GWAS 14 16 ; as a classic inflammation–thrombosis shared gene, ABO governs plasma levels of von Willebrand factor (vWF) and factor VIII, thereby linking genetic variation to both thrombotic risk and inflammatory endothelial activation.

### Genes Associated with Platelet and Endothelial Function

Polymorphisms in platelet membrane glycoprotein genes, such as *ITGB3* (encoding GPIIIa) and *GP1BA* , may alter platelet adhesion and aggregation. 37 Soluble GPVI (shed form of GPVI) predicts AAA growth rate and is a potential therapeutic target. 37 Platelet-derived extracellular vesicles interact with lymphatic endothelial cells to maintain functional integrity. 38 Endothelial function-related genes are also crucial. Polymorphisms in the endothelial nitric oxide synthase ( *eNOS* ) gene affect nitric oxide bioavailability and are associated with a prothrombotic state. 39 Markers of endothelial activation, such as soluble thrombomodulin and vWF, are elevated in AAA patients. 40

### Genes Associated with the Fibrinolytic System

Impaired fibrinolysis promotes persistent ILT accumulation. Tissue-type plasminogen activator (tPA) and urokinase-type plasminogen activator (uPA) are downregulated in endothelial cells and macrophages of AAA lesions, while plasminogen activator inhibitor-1 (PAI-1) is markedly upregulated by inflammatory cytokines (tumor necrosis factor α [TNF-α], interleukin-6 [IL-6]), further suppressing fibrinolytic activity. 41 42 Polymorphisms in the fibrinogen γ-chain ( *FGG* ) gene can alter clot architecture, yielding denser clots resistant to fibrinolysis. 43

### Genes Involved in Inflammation–Thrombosis Crosstalk Regulation

Chronic inflammation and thrombus formation in AAA form a bidirectional crosstalk through key genes and signaling molecules. The nuclear factor κB (NF-κB) pathway serves as a central hub: Upon activation by inflammatory signals, it transactivates prothrombotic genes such as *TF* , *PAI-1* , and *vWF* , initiating coagulation and suppressing fibrinolysis. Conversely, thrombin and activated platelets activate NF-κB via protease-activated receptors (PARs), further upregulating chemokines and adhesion molecules, creating a vicious cycle of “inflammation → thrombosis → intensified inflammation.” As summarized in Table 1 and detailed in previous sections, 41 42 44 45 46 47 48 several signaling pathways serve as molecular bridges between inflammation and thrombosis.

**Table 1:** Key signaling pathways mediating inflammation–thrombosis crosstalk in abdominal aortic aneurysm.

Shared signaling pathway	Core molecules and regulatory mechanisms	Inflammation–thrombosis crosstalk effects	Pathological significance in AAA
NF-κB pathway	Central transcriptional hub activated by TLR4, TNF-α; regulates p65 nuclear translocation	Concurrently upregulates proinflammatory cytokines (IL-6, TNF-α) and procoagulant molecules (TF, vWF)	Drives chronic inflammation and ILT formation, accelerates expansion
Tissue facto pathway	Initiator of coagulation cascade; upregulated by inflammation; forms complex with FVIIa to activate PAR1/PAR2	TF initiates coagulation and activates inflammatory pathways via PARs, upregulating chemokines	Core convergence node of inflammation and coagulation; mediates ILT and inflammatory infiltration
Protease-activated receptor axis	PAR1 and PAR2 activated by thrombin and FXa; couple to MAPK and NF-κB	Converts coagulation signals into inflammatory signals; enhances endothelial permeability, platelet activation, cytokine release	Sustains “coagulation–inflammation” positive feedback loop
ROS–oxidative stress pathway	NOX family enzymes and iNOS mediate excessive ROS generation; suppress eNOS and NO bioavailability	Upstream activation of NLRP3 and NF-κB exacerbates inflammation; enhances platelet aggregation and upregulates PAI-1	Pivotal upstream pathway linking inflammatory activation, endothelial injury, and thrombus formation
JAK–STAT pathway	Activated by IL-6, IFN-γ; STAT1/STAT3 phosphorylation and nuclear translocation	Transduces inflammatory signals to sustain chronic inflammation; upregulates procoagulant genes ( *TF* , *vWF* , *PAI-1* ) and downregulates anticoagulant molecules	Correlated with aneurysm size and ILT burden; potential therapeutic target
NLRP3 inflammasome pathway	Senses oxidative stress and cellular debris; activates Caspase-1 to cleave IL-1β/IL-18	Mediates macrophage pyroptosis and release of proinflammatory cytokines; induces endothelial injury, platelet activation, and TF expression	

Abbreviations: AAA, abdominal aortic aneurysm; eNOS, endothelial nitric oxide synthase; IL, interleukin; ILT, intraluminal thrombus; MAPK, mitogen-activated protein kinase; NF-κB, nuclear factor κB; NOX, NADPH oxidase; PAI-1, plasminogen activator inhibitor-1; PAR, protease-activated receptor; ROS, reactive oxygen species; TF, tissue factor; TNF-α, tumor necrosis factor α; vWF, von Willebrand factor.

### Genes Associated with Hemodynamic Shear Stress and Vascular Wall Remodeling

**Fig. 2:**
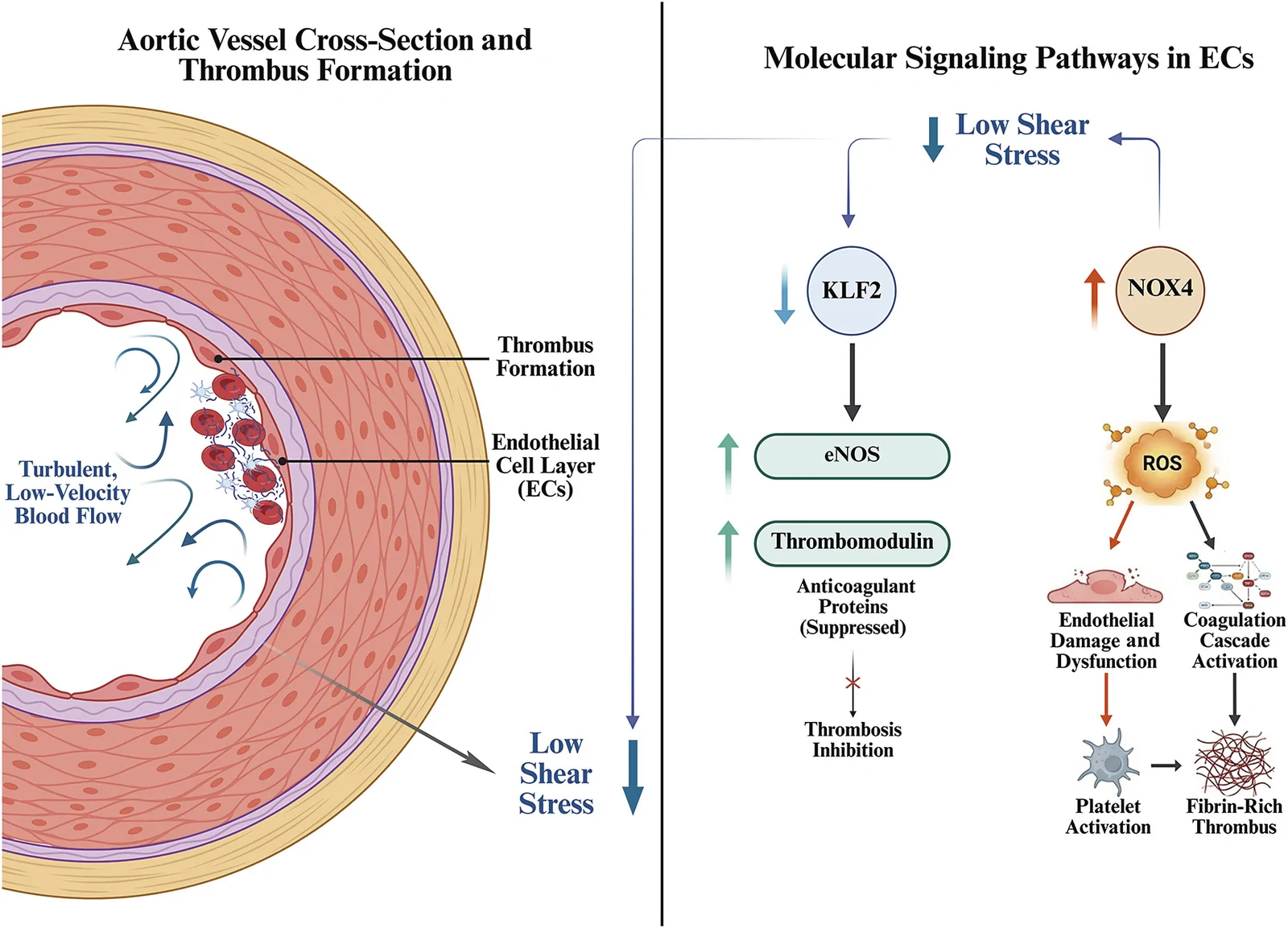
Low shear stress in the aneurysmal sac downregulates KLF2, reducing eNOS and thrombomodulin expression, while upregulating NOX4 to generate ROS. These changes promote endothelial dysfunction, platelet activation, and coagulation, contributing to ILT formation and wall remodeling. (Source: Created in BioRender. Yang Y. (2026). https://app.biorender.com/illustrations/6a119753d85009a59aa1420c?slideId=f9fb10dd-69ea-4cce-bb36-f069db0d274b ). eNOS, endothelial nitric oxide synthase; ILT, intraluminal thrombus; KLF2, Krüppel-like factor 2; NOX4, NADPH oxidase 4.

Hemodynamic abnormalities (low shear stress) within the AAA region further exacerbate thrombus formation by modulating gene expression. KLF2 (Krüppel-like factor 2), a shear stress sensor, is downregulated under low shear stress, failing to induce anticoagulant genes, such as eNOS and thrombomodulin, aggravating the prothrombotic endothelial phenotype. 49 50 51 NADPH oxidase 4 (NOX4) is induced by aberrant shear stress, mediating excessive reactive oxygen species (ROS) generation that both injures endothelium and activates platelets and coagulation pathways ( Fig. 2 ). More recently, the mechanosensitive ion channel PIEZO1 (piezo-type mechanosensitive ion channel component 1, encoded by the FAM38A gene) has been implicated in vascular wall mechanotransduction; endothelial PIEZO1 dysfunction impairs barrier integrity and drives aortic aneurysm and dissection, highlighting PIEZO1 as an emerging mechanosensitive node linking hemodynamic stress to AAA pathology. 52 53

## Shared Signaling Pathways: Integrated Table and Key Nodes

**Fig. 3:**
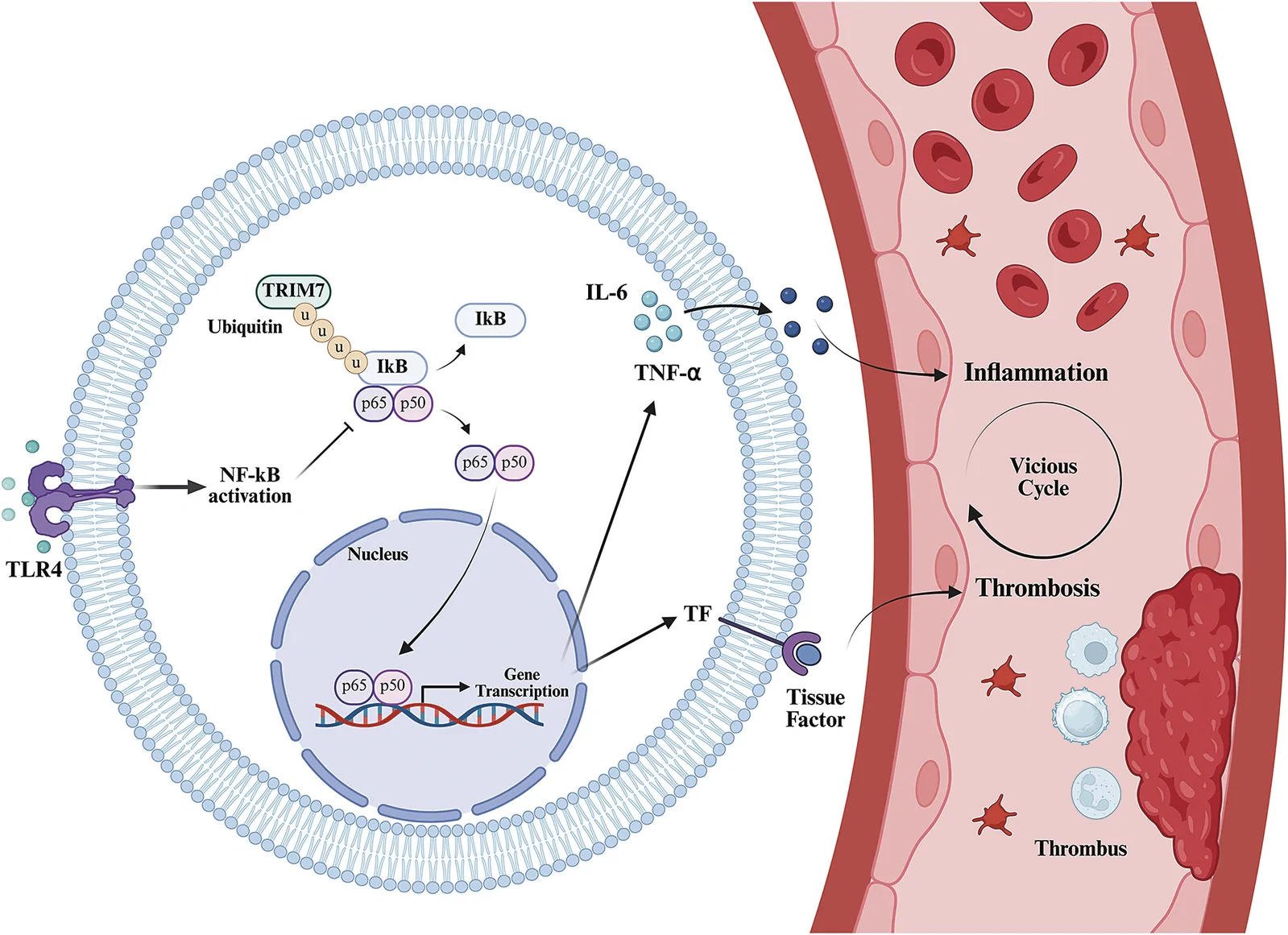
Depicts the NF-κB signaling cascade and its cross-regulation with TF expression. NF-κB activation pathway in AAA. Proinflammatory cytokines (TNF-α, IL-6) and TLR4 ligands activate IKK complex, leading to IκBα degradation and p65 nuclear translocation. Nuclear NF-κB induces transcription of proinflammatory cytokines (IL-6, TNF-α) and procoagulant genes ( *TF* , *PAI-1* , *vWF* ). TF initiates extrinsic coagulation, generating thrombin that activates PARs, which in turn feed back to enhance NF-κB activity. (Source: Created in BioRender. Yang Y. (2026). https://app.biorender.com/illustrations/6a11af3974b999aee5d097e0?slideId=dbee37d7-7246-4df1-a1f2-653c3d1c9cc3 ). AAA, abdominal aortic aneurysm; IL-6, interleukin-6; NF-κB, nuclear factor κB; PAI-1, plasminogen activator inhibitor-1; PAR, protease-activated receptor; TF, tissue factor; TNF-α, tumor necrosis factor α; vWF, von Willebrand factor.

As summarized in Table 1 , several signaling pathways serve as molecular bridges between inflammation and thrombosis. Among them, the NF-κB, TF/PAR, and JAK-STAT axes are the most extensively validated in AAA. The following points highlight their genetic and therapeutic relevance (see Fig. 3 for the NF-κB/TF signaling cascade).

### Risk Prediction Based on Polygenic Risk Scores

PRSs combine the effects of multiple AAA-associated variants into a single estimate of inherited susceptibility. In principle, a score enriched for loci connected to inflammation and thrombosis could provide information beyond aneurysm diameter alone. 54 Available studies suggest that PRSs may help identify people who could benefit from earlier or more intensive ultrasound screening. 55 Evidence for predicting enlargement or rupture is less mature, however, and claims that pathway-restricted scores outperform conventional genome-wide PRSs require prospective head-to-head validation.

Transferability is a major obstacle. A PRS derived mainly from European-ancestry datasets may perform poorly in other populations because allele frequencies, linkage disequilibrium, and estimated effect sizes differ. 55 Evaluation should, therefore, include calibration as well as discrimination, and should be conducted separately in the populations in which the score is intended for use. Age, smoking, hypertension, sex, and family history also need to be modeled with the genetic score rather than treated as competing predictors. Analyses of smoking-related genetic susceptibility illustrate the complexity of separating correlated behavioral and disease pathways. 56 Before PRS-guided screening is adopted, studies must define clinically usable thresholds and show net benefit through decision curve analysis, ideally in prospective cohorts compared with current screening criteria.

### Guiding Individualized Therapy and Pharmacogenomics

Pharmacogenomic information is already used for several cardiovascular drugs, but its relevance to AAA-specific treatment remains uncertain. 57 *CYP2C19* can influence the response to clopidogrel, whereas *CYP2C9* and *VKORC1* affect warfarin dose requirements 58 ; neither example, by itself, establishes that genotype-guided prescribing changes aneurysm growth or rupture risk. A more disease-focused strategy would target nodes that connect inflammation with coagulation, such as NF-κB or TF/PAR signaling. 59 IKKβ (inhibitor of nuclear factor-κB kinase subunit beta; encoded by IKBKB) inhibitors, anti-TF antibodies, and parmodulin-type PAR modulators provide useful experimental examples, but dedicated AAA studies are still needed to establish target engagement, vascular benefit, and bleeding or immunosuppressive toxicity. Multivariable biomarker panels may eventually assist treatment selection, although the incremental value of AI-based models must be tested against simpler clinical models. 57 60 Direct oral anticoagulants inhibit thrombin or factor Xa and may modify protease-dependent PAR signaling; current evidence for favorable vascular effects is largely preclinical and cannot yet justify their use to slow AAA progression in patients without a standard anticoagulation indication. 61

### Epigenetic Regulation and Gene–Environment Interactions

DNA methylation, histone modification, and non-coding RNAs offer one route by which environmental exposure may alter the expression of genetically regulated pathways. 62 Smoking-associated methylation changes are of particular interest in AAA because smoking is a strong clinical risk factor and can affect oxidative stress and immune genes. 63 Epigenome-wide association studies, methylation quantitative trait loci, and expression quantitative trait methylation analyses can connect risk variants with downstream transcriptional effects. 64 65 Interpretation remains difficult: Methylation differences measured after AAA develops may reflect smoking, medication, altered blood cell composition, or the disease itself. Longitudinal sampling and tissue- or cell-specific validation are, therefore, needed before an epigenetic marker can be considered causal, reversible, or clinically useful. 66

### Current Core Challenges and Overall Future Perspectives

Four limitations currently restrict translation. Genetic discovery cohorts remain dominated by participants of European ancestry, so performance in other populations is uncertain. Most associated loci have not been linked to a causal variant, relevant cell type, or experimentally verified mechanism. No standardized pathway-based genetic test has been shown to improve AAA outcomes. Finally, suppressing inflammation–coagulation nodes may weaken host defense or increase bleeding. These are not merely technical details; they determine whether a statistically convincing association can become a safe clinical intervention.

The next phase of the field should prioritize evidence that resolves these limitations. Multiancestry cohorts are needed for discovery and external validation, while fine mapping, perturbation experiments, and cell-specific models should be used to distinguish causal genes from nearby markers. Risk models should be evaluated prospectively against existing screening strategies. For therapeutic studies, enrichment by a relevant genetic or molecular signature may be useful, but only if the signature identifies a biologically coherent subgroup and a measurable treatment response.

## Discussion

The literature reviewed here supports an inflammation–thrombosis component in AAA genetics, but the strength of evidence differs markedly among loci and pathways. GWAS provide robust evidence of association at the population level; single-cell and multiomics studies help nominate cells and mechanisms; and a smaller group of experimental studies tests causality. Keeping these evidence levels separated is important because a shared pathway annotation does not necessarily mean that the same variant causes both inflammation and thrombosis.

## Integration of Genetic Evidence into the Inflammation–Thrombosis Paradigm

Inflammation and thrombosis in AAA are anatomically and biologically connected. Variants near JAK2 and other vascular or immune loci may influence elements of both processes, while TF/PAR and NF-κB signaling provide plausible molecular points of convergence. 14 15 16 Functional studies of *SETDB2, BAF60a* , and interferon-responsive macrophages show how specific perturbations can alter inflammatory signaling, matrix turnover, or aneurysm formation. 6 25 27 The evidence is nevertheless uneven. For several proposed shared genes, the link is based on pathway membership or expression rather than colocalization, allele-specific effects, or direct perturbation of the AAA-associated variant. The term “shared genetic signature” should therefore be interpreted as a working framework, not as proof that every listed locus has dual causal activity.

Genetic overlap between AAA and cardiometabolic traits is consistent with this framework, 18 41 although such correlations cannot identify the tissue or biological process responsible for the overlap. Sex may further modify genetic effects. The cited evidence for sex-dependent hypertension–AAA correlations 33 does not directly test whether NF-κB, TF/PAR, JAK-STAT, or NLRP3 (NOD-like receptor family pyrin domain containing 3; encoded by NLRP3) signaling behaves similarly in men and women. Thus, describing these pathways as sex-independent would be premature; sex-stratified genetic and functional analyses are still required.

## Comparison with Previous Reviews and Meta-Analyses

Earlier AAA genetics reviews summarized susceptibility loci and GWAS findings but gave less attention to how coagulation and inflammatory biology may intersect. 13 14 The distinct contribution of the present review is its organization of evidence around that intersection. This approach is useful for generating testable hypotheses—for example, whether a risk variant changes TF/PAR signaling in a defined vascular cell—but it also exposes an important gap: Many proposed connections remain pathway-level inferences rather than variant-to-function demonstrations.

The broader relationship between inflammation and thrombosis in cardiovascular disease has been reviewed in detail by Stark and Massberg. 41 AAA adds a specific setting in which an organized mural thrombus lies adjacent to a chronically remodeled arterial wall. By combining this anatomical context with genetic and epigenetic findings, 25 64 the current synthesis narrows the question to mechanisms that might explain differences in AAA susceptibility or progression. It does not, however, establish that all thromboinflammatory mechanisms described in other vascular disorders operate in AAA.

## Clinical and Translational Implications

Clinical use of these findings remains prospective. A pathway-informed PRS may prove useful if it predicts clinically important outcomes and adds value beyond age, smoking, family history, and aortic diameter. 12 55 Such superiority has not yet been demonstrated in prospective screening cohorts. NF-κB and TF are attractive experimental targets because each can influence inflammatory and coagulation responses, but systemic inhibition may cause infection, impaired repair, or bleeding. Statins and direct oral anticoagulants have relevant pleiotropic effects, yet existing data do not support prescribing them solely to modify AAA biology. 58 59 SETDB2-mediated H3K9me3 provides an example of a potentially reversible mechanism, 25 although selective delivery and human validation would be required before therapeutic translation.

## Limitations and Gaps in Evidence

This review is limited by the evidence available. Most large genetic datasets represent European-ancestry populations, 14 15 16 20 and both allele frequency and effect size may differ elsewhere. Of the many associated loci, only a small number—including SETDB2 and BAF60a—have substantial functional support in AAA models. 25 27 Environmental modification of genetic effects is biologically plausible but poorly resolved, partly because smoking, diet, medication, and disease stage are difficult to separate in cross-sectional tissue studies. Clinical limitations are equally important: PRS and pharmacogenomic approaches lack standardized thresholds and prospective outcome data. Finally, interventions directed at NF-κB, coagulation, or related shared pathways may trade vascular benefit for immunosuppression or bleeding.

## Future Research Directions

Several studies would directly test the proposed framework. Multiancestry fine mapping could determine whether association signals converge on the same causal variants across populations. Integrating these results with single-cell and spatial transcriptomics could then identify the macrophage, VSMC, endothelial, or platelet states in which a variant is active. Candidate mechanisms should be tested by perturbing the implicated variant or gene in relevant cells and AAA models rather than relying on expression correlation alone. In parallel, an AAA PRS should be compared prospectively with current screening criteria and evaluated for calibration, discrimination, and clinical net benefit. Local delivery of agents targeting NF-κB or TF/PAR signaling may reduce systemic toxicity, but efficacy and bleeding outcomes must be measured together. Machine learning models may assist integration of genetic, epigenetic, and clinical variables; transparent comparison with simpler models will be necessary to show that added complexity is justified.

## Conclusion

AAA develops through interacting changes in the arterial wall, immune compartment, hemostatic system, and ILT. Genetic studies implicate pathways that can influence more than one of these processes, with NF-κB, TF/PAR, and JAK-STAT signaling emerging as plausible points of convergence. The present evidence is strongest for association and pathway enrichment; causal variants, relevant cell types, and clinical utility remain unresolved for most loci. Progress therefore depends on multiancestry fine mapping, direct functional testing, and prospective evaluation of genetic models alongside established clinical risk factors. Any therapy directed at a shared inflammatory–thrombotic node will also need to demonstrate that vascular benefit outweighs bleeding and immune-related harm. Until those conditions are met, these genetic signatures are best viewed as a guide for mechanism-based research rather than a basis for routine AAA management.

## Data Availability

This review is based on previously published studies. No new data were generated or analyzed. All referenced data can be accessed through the cited publications.
